# Comprehensive Evaluation of Coal-Fired Power Units Using Grey Relational Analysis and a Hybrid Entropy-Based Weighting Method

**DOI:** 10.3390/e20040215

**Published:** 2018-03-23

**Authors:** Dianfa Wu, Ningling Wang, Zhiping Yang, Chengzhou Li, Yongping Yang

**Affiliations:** National Research Center for Thermal Power Engineering and Technology, North China Electric Power University, Changping District, Beijing 102206, China

**Keywords:** coal-fired power units, GRA, bootstrap, entropy, AHP, sensitivity analysis

## Abstract

In recent years, coal-fired power plants contribute the biggest part of power generation in China. Challenges of energy conservation and emission reduction of the coal-fired power plant encountering with a rapid growth due to the rising proportion of renewable energy generation in total power generation. Energy saving power generation dispatch (ESPGD) based on power units sorting technology is a promising approach to meet the challenge. Therefore, it is crucial to establish a reasonable and feasible multi-index comprehensive evaluation (MICE) framework for assessing the performance of coal-fired power units accessed by the power grid. In this paper, a hierarchical multiple criteria evaluation system was established. Except for the typical economic and environmental indices, the evaluation system considering operational flexibility and power quality indices either. A hybrid comprehensive evaluation model was proposed to assess the unit operational performance. The model is an integration of grey relational analysis (GRA) with analytic hierarchy process (AHP) and a novel entropy-based method (abbreviate as BECC) which integrates bootstrap method and correlation coefficient (CC) into entropy principle to get the objective weight of indices. Then a case study on seven typical 600 megawatts coal-fired power units was carried out to illustrate the proposed evaluation model, and a weight sensitivity analysis was developed in addition. The results of the case study shows that unit 4 has the power generating priority over the rest ones, and unit 2 ranks last, with the lowest grey relational degree. The weight sensitivity analysis shows that the environmental factor has the biggest sensitivity coefficient. And the validation analysis of the developed BECC weight method shows that it is feasible for the MICE model, and it is stable with an ignorable uncertainty caused by the stochastic factor in the bootstrapping process. The elaborate analysis of the result reveals that it is feasible to rank power units with the proposed evaluation model. Furthermore, it is beneficial to synthesize the updated multiple criteria in optimizing the power generating priority of coal-fired power units.

## 1. Introduction

In recent years, increased electricity consumption demand in China has been restricted by the “new normal” economic situation. At the generation side of the power grid, the share of installed renewable energy capacity has achieved to 38.1 percent of total power generating [1]. Thus, the utilization hours of thermal power plants were less than the previous years and the coal consumption was higher than the long-term low-load operation design condition. The soaring development of renewable energy, indeed, is beneficial to the energy saving and emission reduction for the whole national energy generation system. However, due to the random, unstable and hard to predict characteristics of the renewable energy, more peak shaving resources are required. Unfortunately, peaking power capacity in China such as pumped-hydro energy storage stations, gas-fired power stations and energy storage is too small to meet the rapid demand [2]. As coal-fired power plants are still the main suppliers of electricity, therefore, taking full use of peak shaving potentiality of the typical thermal power unit is the most practical way to meet the challenge [3,4].

For the power grid, ESPGD is one of the promising ways to achieve the optimal allocation of various energy generation sources based on coal-fired power units [5,6,7,8]. It is an essential task to determine the ESPGD priority list of the coal-fired units available to the grid. The traditional evaluation method concerns only economic index such as coal consumption rate which is usually used as equal incremental principle in the power dispatching system, and for different power units, the power generation and utilization hours are allocated in equally [9]. In this way, all the power generating facilities have the same generation rights and hours ignoring the power unit’s efficiency. Thus, this causes huge energy and resource waste and serious environmental pollution. The Energy Saving Power Generation Dispatching Measures (trial), distributed by National Development and Reform Commission (NDRC) on 2 August 2007, taken into account the environmental indices in addition to the economic indicators [5]. However, the operational flexibility of coal-fired power units is becoming more and more important for the power grid peak shaving [2,10,11]. Moreover, electricity, is a kind of industrial product of the power generator, whose quality such as frequency, voltage indices should be considered, especially for the peak shaving process. Therefore, the evaluation indices of coal-fired power units need to be expanded.

To determining the priority of candidate power unit with multiple performance indices, some prevalent multi-criteria decision making (MCDM) methods are available for the typical complex system, such as PROMETHEE [12], ELECTRE [13], TOPSIS [14,15,16], grey theory [17], Fuzzy method [18], AHP [19] and entropy method [20,21], etc. It is a widely accepted technology that people usually combine various classical MCDM methods to acquire integrated advantages [14,15,22,23,24,25,26]. Grey system theory has been widely used in decision making or comprehensive evaluation problems [4,17,22,26,27]. For many times, the mechanism of raw data we faced is not deterministic and partially known. However, the grey model only requires a limited amount of data to estimate the behavior of unknown systems and can derive an unbiased estimate.

In the process of comprehensive evaluation, the importance of the index is usually different for the decision maker. To obtain the weights of the indices, some methods have been introduced in literatures such as the subjective, objective and combination methods. The subjective preference of experts can be determined by the method such as Delphi [28], AHP [4,15,16,26,29,30], etc. The objective weighting method, which derived only from the evaluating data set, contains entropy [20,21,31], DEA [32], CRITIC [33] and CCSD [34], etc. The combination weights can make a compromise of the subjective weight and the objective weight. The AHP method is a powerful tool in making complicated and often irreversible decisions benefited from its ability of decomposing a complex problem into multiple layers and the capacity of quantitatively treating complex and multi-criteria systems, and the entropy method is one of the most popular objective weighting methods. However, the classical entropy weighting method did not concern the interrelation of each criterion like the CRITIC [33] and CCSD [34] weighting method, these two methods are similarly integrated correlation coefficient and standard deviation [34,35] of evaluation indices from deferent perspectives. In sometimes, the comprehensive evaluation results may vary notably with different index weights achieved from subjective weighting method. Thus, it is necessary to carry out weight sensitivity analysis [27,34,36], which can perform a quantitative study of the detailed weight information. Additionally, the data used for the comprehensive evaluation or decision making collected from a population usually has a small sample size. Bootstrap developed by Efron is an optional way to maximize utilizing of the population information hidden in the samples [37,38,39]. This resampling method is typically used in non-parametric estimation, no matter what the population distribution is.

In this paper, for assessing the comprehensive performance of coal-fired power units, we propose a multiple index evaluation system considering economic, environmental, flexibility and power quality criteria. A novel integrating objective weighting method (BECC) was developed by combining bootstrap method, entropy and correlation coefficient weighting method. The rest of the paper is organized as follows: in Section 2, evaluation indices system is introduced. Section 3 introduces the hybrid methodology with GRA and combined weighing method using AHP and the proposed BECC method. Section 4 discusses a case study to illustrate the effectiveness of the evaluation model for power units. Section 5 shows the results discussion and model validation, Section 6 draws some conclusions. The symbols used in this paper are listed in the Nomenclature section.

## 2. Performance Indices of Power Units

The choice of the index is very important for any successful comprehensive evaluation work. The selected indices must serve the purpose of the evaluation. Usually, the following principles should be followed:

- *Integrity*. Indices should be able to reflect the power grid requirements to the electricity generating facilities in an accurate and objective way according to professional theory or prior knowledge.
- *Independence*. Because of the complex relationships between indicators, the indices selected should be the one with less redundancy at the precondition of integrity.
- *Availability*. The index should be reliable and can be accessed easily in the current technical conditions.

**Figure 1:**
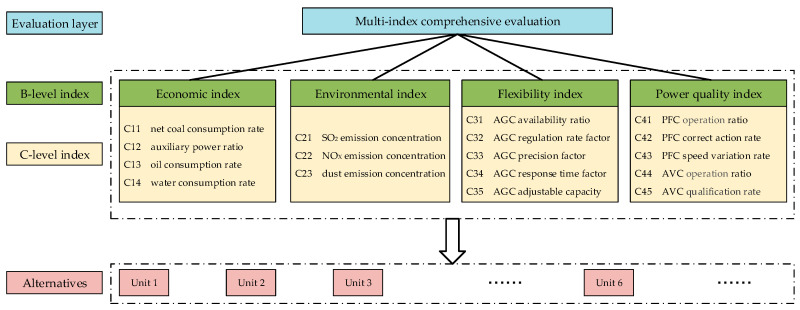
Hierarchy of multi-level index evaluation system.

We take four categories into consideration for the comprehensive evaluation task. The index Hierarchy, shown in Figure 1, is comprised of economic index (B1), environmental index (B2), flexibility index (B3) and power quality index (B4). The four B-level indices decompose C-level subindices respectively. The detailed C-level indices are introduced as follows.

### 2.1. Economic Index

The economic index contains net coal consumption rate (C11), auxiliary power ratio (C12), oil consumption rate (C13) and water consumption rate (C14). Coal consumption rate is one of the most important indices representing power plant performance; the rate represents the amount of standard coal consumed per kWh. The auxiliary consumption rate reflects the electric power consumption of the power unit itself. The oil consumption is caused by the processes of unit startup-shutdown and the low-load combustion (for the flame stability of boiler). The water consumption rate reflects the water saving benefits. For these four indices, the smaller the better.

### 2.2. Environmental Index

We primarily consider the gaseous pollutant in the coal-fired boiler flue gas exhaust. That is, SO_2_ emission concentration (C21), NO_X_ emission concentration (C22) and dust emission concentration (C23). The emission of Hg pollutant and greenhouse gases such as CO_2_ are not considered mainly because such pollutant removal equipment has not been widely used in China. Pollutant emissions from coal burning are a major contributor to environmental pollution, these three indices reflect the ability of environment protection of the power unit. For these three indices, the smaller the better. All these indicators are available in the online monitoring platforms at current plants.

### 2.3. Flexibility Index

Flexibility includes fuel flexibility and operational flexibility. In this work, the indices we used only refers to operational flexibility. The flexibility requirements of the power grid system for power units mainly contain the aspects of peak adjustment depth, peak regulation speed and the ability of quick startup-shutdown, etc. Referring to standard codes of the Detailed Rules for The Implementation of Auxiliary Service Management of Power Plant Connected in North China Grid (Trial) and the Detailed Rules for The Implementation of Grid Operation Management in North China Regional Power Plant (Trial) (“Two Rules” published by North China Branch of State Electricity Regulatory Commission, 2009), five indicators of Automatic Generation Control (AGC) performance are selected in our proposed multi-level index evaluation system for characterizing the flexibility features. The AGC indices can be obtained from the Supervisory Control and Data Acquisition (SCADA) platform, which is a subsystem of the Energy Management System (EMS) used in the power grid. The data are averaged on an annual level. The five indices are introduced as follows.

AGC availability ratio (C31) is the ratio of AGC input time to the effective running time during the statistical period. AGC regulation rate factor (C32) is the ratio of the regulating rate to the standard regulating rate of the electric power generation unit. The regulating rate is the average of the rising rate and the decline rate in the statistical period. AGC regulation precision factor (C33) is the ratio of the regulation deviation to the allowable deviation. The regulation deviation is the value between the AGC load instruction with the actual stable output of generator responding. AGC response time factor (C34) is the ratio of actual response time to the standard response time. The AGC response time is used to break through the regulation dead zone in the same adjustment direction with the EMS signal based on the original generator output point. AGC adjustable capacity (C35) refers to the ratio of the adjustable unit capacity for the power grid to the design load rate.

### 2.4. Power Quality Index

For a coal-fired power generator, the power quality control mainly refers to the regulation of voltage quality and frequency quality. The adjustment ability of power units can be reflected by the indices from the primary frequency control (PFC) and automatic voltage control (AVC) systems. The power quality indices contain five C-level indices.

PFC operation ratio (C41) is the ratio of PFC running time to the power unit operation time available to the grid. PFC correct action rate (C42) refers to the ratio of the PFC correct action number to the total adjustment times during the statistical period. PFC speed variation rate (C43) is the ratio of the static speed increase to the rated speed. The static speed increase happens in the process of power load varying from the rated load to the empty load. For the unit, the greater the index value, the smaller the contribution to the grid peak shaving. AVC operation ratio (C44) is the ratio of AVC running time to the power unit operation time available to the grid. AVC qualification ratio (C45) is the ratio of the AVC correct action times to the total adjustment times during the statistical period.

## 3. Methodology of Multi-Index Comprehensive Evaluation

In this section, a hybrid evaluation model was developed for coal-fired power units. The model integrates the GRA theory, AHP weighting method and a proposed entropy-based objective weighting method (BECC). The subsection provides an overview of some key theories including sensitivity analysis method and finally illustrates the integrated algorithm.

### 3.1. An Overview of GRA Evaluation Method

Grey system theory has been widely used in various fields of science because of its advantages in evaluating complex systems with various correlated indicators, and the GRA method [4,17,26] is developed based on the grey system theory. The general steps for the evaluation are five steps, i.e., indices determination, data dimensionless processing, evaluation matrix determination, weight determination of each index and the comprehensive evaluation.

The grey relational theory determines the correlation degree according to the similarity among the sequence curves, i.e., the more similarity between two sequences, the higher the correlation degree will be. Therefore, in each index layer, we assume an ideal or optimal index sequence as a reference and then consider the similarity of the indices between the evaluated alternatives and the reference sequence. The more similarity between the two index sequences, the better the comprehensive performance of the alternatives investigated. Therefore, when evaluating a system using the GRA method, an ideal alternative with optimal indices should be introduced first as a reference sequence, then the grey relational coefficients between the reference sequence and each alternative are calculated to establish the grey relational coefficient matrix.

#### 3.1.1. Normalizing the Evaluation Indices

For m kind evaluation indices $f_{i}\left(1\leq i\leq m\right)$, and n kind alternatives $a_{j}\left(1\leq j\leq n\right)$, they construct a sample matrix $X={\left(x_{ij}\right)}_{n\times m}$, X can be written as:

(1) $$X={\left(x_{ij}\right)}_{n\times m}=\begin{array}{c} a_{1}\\ a_{2}\\ \vdots \\ a_{n} \end{array}\;\overset{\begin{array}{cccc} f_{1}\; & f_{2}\; & \;\cdots \; & f_{m} \end{array}}{\left[\begin{array}{cccc} x_{11} & x_{12} & \cdots & x_{1m}\\ x_{21} & x_{22} & \cdots & x_{2m}\\ \vdots & \vdots & \ddots & \vdots \\ x_{n1} & x_{n2} & \cdots & x_{nm} \end{array}\right]}$$

The indices usually have different dimensions and magnitudes. Therefore, the normalizing procedure may be carried out with the indices. For the index which is the bigger the better (or benefit attributes), it can be normalized as:

(2) $$y_{ij}=\frac{x_{ij}-\underset{i}{\min}(x_{ij})}{\underset{i}{\max}(x_{ij})-\underset{i}{\min}(x_{ij})},(1\leq i\leq n,1\leq j\leq m)$$

and for the index which is the smaller the better (or cost attributes), it can be normalized as:

(3) $$y_{ij}=\frac{\underset{i}{\max}(x_{ij})-x_{ij}}{\underset{i}{\max}(x_{ij})-\underset{i}{\min}(x_{ij})},(1\leq i\leq n,1\leq j\leq m)$$

where in the Equations (2) and (3), $\underset{i}{\max}\left(x_{i,j}\right)$ and $\underset{i}{\min}\left(x_{i,j}\right)$ mean the maximum and the minimum value in row i and $Y={\left(y_{ij}\right)}_{n\times m}$ is called the linear scale standardized matrix.

#### 3.1.2. Calculating the Grey Relational Coefficient Number

According to the GRA theory, set $y^{ref}=\left\{y^{ref}\left(1\right),y^{ref}\left(2\right),\;\ldots ,y^{ref}\left(m\right)\right\}$ as the reference sequence. The series is the comparing one. Using the GRA can get the coefficient number about the jth index of the ith alternative. The matrix can be written as:

(4) $$Z={\left(z_{ij}\right)}_{n\times m}\;=\left|y_{j}^{ref}-y_{ij}\right|,\;(1\leq i\leq n,1\leq j\leq m)$$

and then the grey relational coefficient matrix is obtained, with the elements formulated as:

(5) $$\xi_{ij}=\frac{\underset{i}{\min}\underset{j}{\min}\{z_{ij}\}+\rho \underset{i}{\max}\underset{j}{\max}\{z_{ij}\}}{z_{ij}+\rho \underset{i}{\max}\underset{j}{\max}\{z_{ij}\}},\;(1\leq i\leq n,1\leq j\leq m)$$

where $\xi_{ij}$ is the grey relational coefficient of the jth index of the ith alternative. The factor ρ∊[0, 1] is the distinguishing coefficient and usually set to 0.5.

#### 3.1.3. Calculating Grey Relational Result Vector

Based on the methodology of GRA and the indices weights $W={\left(w_{1},w_{2},\ldots ,w_{m}\right)}^{T}$, the final calculation model can be deduced as:

(6) $$V=\xi \cdot W={\left(v_{1},v_{2},\ldots v_{n}\right)}^{T}$$

where $v_{i}=\overset{m}{\underset{j=1}{\sum }}\xi_{i}\left(j\right)\cdot w_{j},\;\left(1\leq i\leq n,\;1\leq j\leq m\right)$, the vector V is the result of the evaluated alternatives, and ξ is the grey relational coefficients matrix of indices, W is the weight of the evaluated indices. According to the principle of maximum correlation, the evaluation alternative can be sorted, the larger the $v_{i}$, the better the alternative.

### 3.2. Objective and Subjective Weighting Method

#### 3.2.1. Proposed BECC Objective Weighting Algorithm

It is significant to utilize the sample information sufficiently in calculating objective weight, because the algorithm is only based on the intrinsic structure of the selecting sample data. Entropy principle is a well-known tool to quantify the uncertainty of raw data. Besides, the influence of the correlation between indicators should be considered either at the same time. On the other hand, the size of the sample we faced is usually too small as the difficulty of collecting data. Thus, based on entropy principle, the CC weight method and bootstrap resample simulation approach are integrated into BECC method.

##### A. An Overview of the Bootstrap Method

Bootstrap [37,39] is the most common estimation method, which can make full use of the information contained in the samples, which especially suits for small samples. The key idea of the bootstrap method is to estimate the sampling distribution by the resampling asymptotic relative frequency distribution instead of the population distribution in estimating parameters.

For a statistical sample sequence $X=\left(x_{i}\right),\;i=1,2,\ldots ,n$, the estimated parameter $\hat{\theta }$ of the sample can be calculated by the following steps:

Step 1. An empirical probability distribution $\hat{F}\left(x\right)$ is built up firstly, which is a empirical distribution function of x, the distribution $\hat{F}\left(x\right)$ is the maximum likelihood estimate of the population distribution function F(x).
Step 2. Generating a resampled sequence $X_{b}^{*}=\left(x_{bi}^{*}\right),\;i=1,2,\ldots ,n$, $x_{b}^{*}$ is generated from the empirical distribution $\hat{F}\left(x\right)$ by the method of random sampling with replacement.
Step 3. Calculating the estimated parameter $\hat{\theta }$ of the resampled sequence, we get ${\hat{\theta }}_{b}^{*}$ based on bootstrap method.
Step 4. Repeating Step 2 and Step 3 for B times (usually more than 1000 times).
Step 5. Constructing a new estimate distribution based on the resampled data sequence ${\hat{\theta }}_{b}^{*},\;b=1,2,\ldots ,B$ produced by Step 4. The bootstrap distribution ${\hat{F}}^{*}\left({\hat{\theta }}^{*}\right)$ can be used for the estimation of the parameter $\hat{\theta }$.

##### B. An Overview of Entropy Weighting Method

Entropy is initially a concept of thermodynamics, which is used to describe an irreversible phenomenon of the motion state, and later in the information field, it is used to measure the uncertainty of things. The entropy weight is calculated out by the judgment matrix [18,20,31]. From the point of information, entropy provides measurement instruments for the amount of the useful information provided in the problem. The greater the amount of information has, the less the uncertainty and the entropy are, and vice versa. The steps of the method to determine the weight are given in the following briefly.

For the decision matrix X of n kind alternatives and m kind evaluation indices, which formulated as:

(7) $$X={\left(x_{ij}\right)}_{n\times m},\;i=1,2,\ldots ,n;\;j=1,2,\ldots ,m$$

The first step, normalizing the matrix:

(8) $$p_{ij}=x_{ij}/\sum_{i=1}^{n}x_{ij},\;i=1,2,\ldots ,n;\;j=1,2,\ldots ,m$$

The second step, calculating the entropy of each index:

(9) $$e_{j}=\frac{\sum_{i=1}^{n}p_{ij}\ln p_{ij}}{\ln n}$$

The third step, calculating the entropy weight value:

(10) $$w_{j}^{ENT}=\frac{1-e_{j}}{\sum_{j=1}^{m}\left(1-e_{j}\right)},\;j=1,2,\ldots ,m$$

where the superscripts “ENT” of $w_{j}^{ENT}$ means entropy method based.

##### C. An Overview of the Correlation Coefficient Objective Weighting Method

The linear correlation coefficient (CC) [34] between the index vectors ($f_{j}$) of the evaluation data depicted in Formula (1) is a symmetric matrix, with the dimension of m×m and a generic element of $r_{jk}$, that is $R={\left(r_{jk}\right)}_{m\times m}$, where $r_{jk}$ is calculated by:

(11) $$r_{jk}=\frac{\sum_{i=1}^{n}\left(z_{ij}-{\bar{z}}_{j})(z_{ik}-{\bar{z}}_{k}\right)}{\sqrt{\sum_{i=1}^{n}{\left(z_{ij}-{\bar{z}}_{j}\right)}^{2}\cdot \sum_{i=1}^{n}{\left(z_{ik}-{\bar{z}}_{k}\right)}^{2}}},\;j,k=1,\cdots ,m.$$

We use the sum vector $\overset{m}{\underset{k=1}{\sum }}\left(1-r_{jk}\right)$ that represents a measure of the conflict created by index vector $f_{j}$ with respect to the rest indices. That denotes the more discordant scores of the alternatives in criteria $f_{j}$ and $f_{k}$, the lower value of $r_{jk}$ should be assigned. Then normalizing the sum vector, CC weight can be got with the formula:

(12) $$w_{{}_{j}}^{CC}=\frac{\sum_{k=1}^{m}\left(1-r_{jk}\right)}{\sum_{j=1}^{m}\sum_{k=1}^{m}\left(1-r_{jk}\right)},\;j=1,\cdots ,m.$$

##### D. Integrated BECC Objective Weighting Method

This subsection introduces the integrating algorithm of BECC in details. Setting the entropy and CC weights as the estimating parameters in the bootstrap procedure (see A), resample matrices of entropy (see B) and CC (see C) weights with the dimension of B×m can be obtained respectively. That is, in each time of resample, new weight vectors of $W^{ENT}$ and $W^{CC}$ will be calculated. Then bootstrap-entropy and bootstrap-correlation coefficient weights denoted as $W^{BENT}$ and $W^{BCC}$ can be got by the averaging method, which formulated as:

(13) $$w_{j}^{BENT}=\frac{\sum_{b=1}^{B}w_{bj}^{ENT}/B}{\sum_{j=1}^{m}(\sum_{b=1}^{B}w_{bj}^{ENT}/B)},\;j=1,\cdots ,m.$$

(14) $$w_{{}_{j}}^{BCC}=\frac{\sum_{b=1}^{B}w_{bj}^{CC}/B}{\sum_{j=1}^{m}(\sum_{b=1}^{B}w_{bj}^{CC}/B)},\;j=1,\cdots ,m.$$

Due to the difference between the largest and the smallest CC weights element is outstanding, root squared method is employed to shorten the difference [34]. Based on the above analyses, we define the BECC weights of attributes as:

(15) $$w_{j}^{BECC}=\frac{w_{j}^{BENT}\cdot \sqrt{w_{{}_{j}}^{BCC}}}{\sum_{j=1}^{m}w_{j}^{BENT}\cdot \sqrt{w_{{}_{j}}^{BCC}}}$$

**Figure 2:**
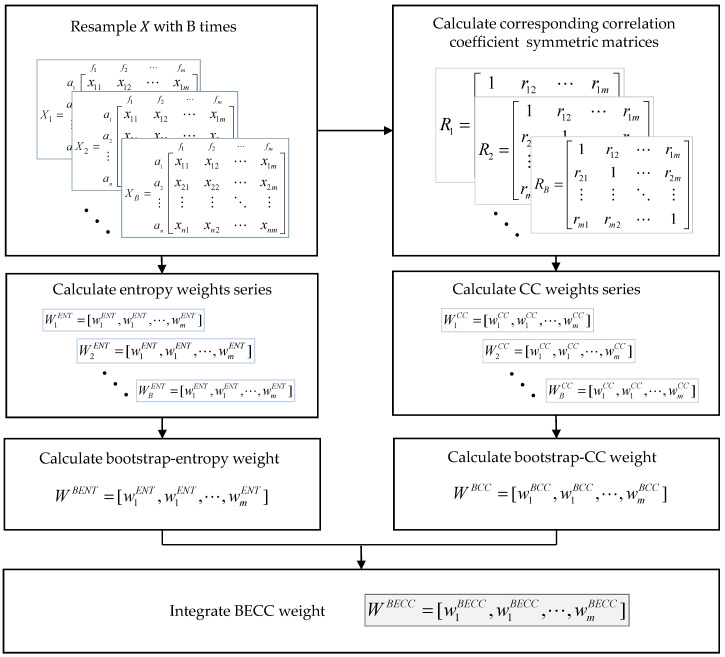
Steps of BECC weight calculation algorithm.

The BECC weight calculation algorithm is shown in Figure 2.

#### 3.2.2. An Overview of AHP Weighting Method

The AHP method [4,19,26] reflects the thinking steps of the decision maker, that is, decomposition, judgment and synthesizing. First, a hierarchical decision system is formed according to the characteristics of the evaluation alternatives, the weights of different indices can be determined by pairwise comparison process. The measurement scale of 1 to 9 is used to represent such relative importance (see Table 1). The brief steps are as follows.

First, the pairwise comparison matrix $A={\left(a_{jk}\right)}_{m\times m}$ is got by the method mentioned above from the decision maker.

Then we standardize the comparison matrix A using the equation:

(16) $${\bar{a}}_{jk}=a_{jk}/\sum_{k=1}^{m}a_{jk},\;(j,k=1,2,\cdots ,m)$$

For the normalized matrix $\bar{A}={\left({\bar{a}}_{jk}\right)}_{m\times m}$, we sum the elements of the matrix $\bar{A}$ as rows, and then get the weight vector:

(17) $${\bar{w}}_{j}=\sum_{k=1}^{m}{\bar{a}}_{jk},\;(i=1,2,\cdots ,m)$$

To validate the decision of the AHP, the following steps are developed. Consequently, normalizing the matrix composed of ${\bar{w}}_{j}$:

(18) $$w_{j}=\frac{{\bar{w}}_{j}}{\sum_{j=1}^{m}{\bar{w}}_{j}},(j=1,2,\cdots ,m)$$

then we gain the AHP weight $W^{AHP}=\left(w_{1},w_{2},\ldots ,w_{m}\right)$, where $\overset{m}{\underset{j=1}{\sum }}w_{j}=1$.

The maximum eigenvalue $\lambda_{\max}$ is calculated by the formula:

(19) $$\lambda_{\max}=\frac{1}{m}\sum_{j=1}^{m}\frac{{\left(AW\right)}_{j}}{w_{j}}$$

Using the criteria in Table 2, consistency ratio (*CR*) is carried out to check the consistency:

(20) CR=CI/RI

where $CI=\left(\lambda_{\max}-m\right)/\left(m-1\right)$ (*m* is the dimension of the comparison matrix) and *RI* is the random index.

Finally, if the value CR<0.1, the results are assumed passed through the consistency check, otherwise, we need to adjust the pairwise comparison matrix and recalculating until the results passed through the consistency check.

#### 3.2.3. Integrating BECC and AHP Weights with Product Method

It is reasonable to combine two kinds of weighs to take full performance advantage of objective weight (by BECC method) and subjective weight (by AHP method), the two type weight are combined to generate a comprehensive weight, the following production method should be used. It terms to be:

(21) $$w_{j}=(w_{j}^{BECC}\cdot w_{j}^{AHP})/\sum_{j=1}^{n}(w_{j}^{BECC}\cdot w_{j}^{AHP}),\;j=1,2,\ldots ,m$$

where the $w_{j}^{BECC}$ and $w_{j}^{AHP}$ are the BECC and AHP weight of each index, respectively.

### 3.3. The Hybrid Model for Comprehensive Evaluation

A hybrid model is developed with the methods mentioned in Section 3.1 and Section 3.2 to give a sensible comprehensive evaluation of coal-fired power units connected to the power grid system. For the multiple index evaluation system given in Figure 1, the assessment procedure is carried out mainly by two steps.

Firstly, C-level evaluation is implemented by GRA with BECC weighting method. In this step, only objective weight is used in exploiting the C-level indices data, the AHP method is not used. The reason for this strategy is illustrated as follows. The AHP method, which needs a lot of expert knowledge to fulfill the pairwise comparison matrix (Section 3.2.2), usually cost a very expensive practice in deciding the priory of the complex bottom indices (C-level index). As a matter of fact, the expert is easier to give a reasonable decision value to the high-level index than to a bottom one, and for the indicators in a high-level category, it is easier to pass through the AHP consistency check as the fewer indices numbers. Based on the above reasons, we only fuse the expert knowledge by AHP weights in the B-level evaluation process. In this way, expert preference is ultimately reflected in the final evaluation result.

**Figure 3:**
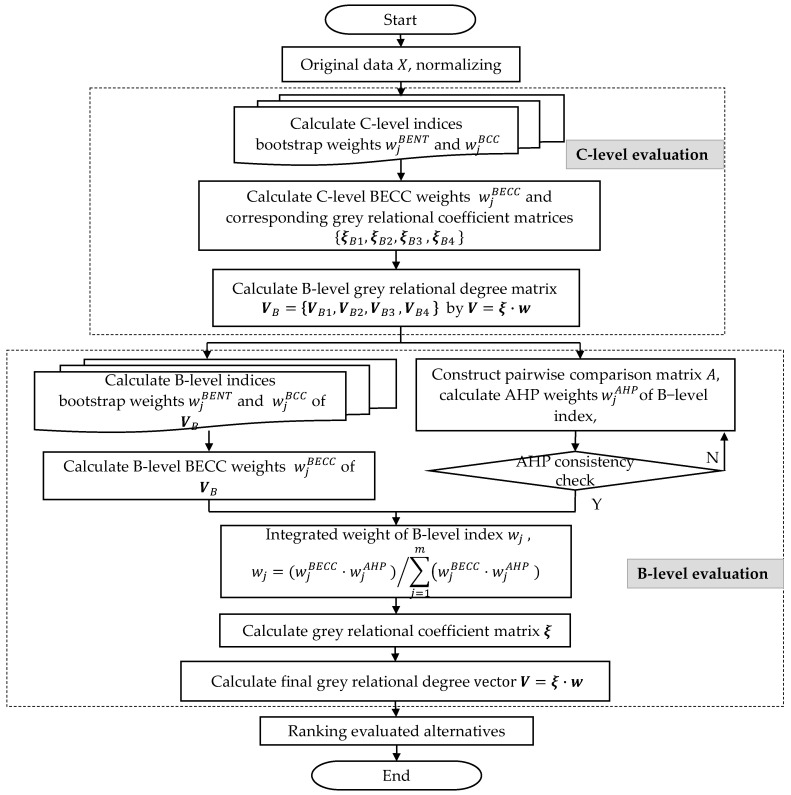
The flowchart of the comprehensive evaluation algorithm.

Secondly, grey relational evaluation vectors of each B-level index from the C-level evolution step by Equation (6) constructs a B-level decision matrix. For this matrix, BECC method is implemented again and the AHP method is also used in this step to get a combined weight W by the Formula (21). The final evaluation vector can be obtained by the integration of GRA and the weight W using the Equation (6) again. The flowchart of this hybrid multiple index comprehensive evaluation model is illustrated in Figure 3.

### 3.4. Weighting Sensitivity Analysis of Evaluation Indices

The stability of evaluation results is an important aspect that should be taken into account with the multiple index comprehensive evaluation problem. The sensitivity analysis is used to produce complementary information to the evaluation process. The weight sensitivity was theoretically analyzed under the assumption that every index of the alternatives was perturbed with the same ratio while the other indices kept unchanged. The sensitivity analysis result can draw out the supplementary conclusion with the robustness of the evaluation results. The weights with the high sensitivity should be got particular attention because the evaluation results will easier vary with a little weight change. The following approach is employed to quantify the weight sensitivity [27].

For the evaluation matrix shown in Equation (1), if the ranking result of alternatives $a_{i},a_{k}\in A$ inversed with the weight changes $\varphi_{h,i,k}\left(1\leq i,k\leq n,1\leq h\leq m\right)$ in the hth index, then $\varphi_{h,i,k}$ is defined as an absolute minimum change of the hth index weights, and $\varphi_{h,i,k}^{\prime }=\varphi_{h,i,k}/w_{h}\times 100\%$ is the relative minimum change.

The varied weight $w_{h}^{*}$ is denoted as $w_{h}^{*}=w_{h}-\varphi_{h,i,k},\;0<w_{h}^{*}<1$. Normalizing $w_{h}^{*}$ deduces the new perturbed weights formulated as:

(22) $$w_{h}^{\prime }=\frac{w_{h}^{*}}{\sum_{j=1}^{h-1}w_{j}+w_{h}^{\prime }+\sum_{j=h+1}^{n}w_{j}}$$

(23) $$w_{j}^{\prime }=\frac{w_{j}}{\sum_{j=1}^{h-1}w_{j}+w_{h}^{\prime }+\sum_{j=h+1}^{n}w_{j}},\;1\leq j\leq n,j\neq h$$

Supposing the evaluation value $V_{i}>V_{k}$ of alternatives $a_{i},a_{k}\in A$, $V_{i}^{\prime }$ and $V_{k}^{\prime }$ are the new corresponding evaluation results after the hth index weighing changed. When the inverse ranking results happened, i.e., $V_{i}^{\prime }<V_{k}^{\prime }$, the formula is:

(24) $$\sum_{j=1}^{n}w_{j}^{\prime }h_{i,j}<\sum_{j=1}^{n}w_{j}^{\prime }h_{k,j}$$

The inequality expression can be got from Equations (22)–(24):

(25) $$\sum_{j=1}^{n}w_{j}h_{i,j}-\varphi_{h,i,k}h_{i,h}<\sum_{j=1}^{n}w_{j}h_{k,j}-\varphi_{h,i,k}h_{k,h}$$

Considering that $V_{i}=\overset{n}{\underset{j=1}{\sum }}w_{j}h_{i,j}$ and $V_{k}=\overset{n}{\underset{j=1}{\sum }}w_{j}h_{k,j}$, thus, it is turned out as:

(26) $$V_{i}-V_{k}<\varphi_{h,i,k}(h_{i,h}-h_{k,h})$$

Here we got the final absolute minimum change $\varphi_{h,i,k}$ using the terms:

(27) $$\varphi_{h,i,k}>\frac{V_{i}-V_{k}}{h_{i,h}-h_{k,h}},\;h_{i,h}>h_{k,h}$$

(28) $$\varphi_{h,i,k}<\frac{V_{i}-V_{k}}{h_{i,h}-h_{k,h}},\;h_{i,h}<h_{k,h}$$

Due to the changed weights $w_{h}^{\prime }$ is also constrained by the relation expression:

(29) $$0<w_{h}^{\prime }=w_{h}-\varphi_{h,i,k}<1$$

thus, the $\varphi_{h,i,k}$ is subjected to the condition of $w_{h}-1<\varphi_{h,i,k}<w_{h}$ at the same time.

While the corresponding relative weight change $\varphi_{h,i,k}^{\prime }$ is calculated by the following formulas:

(30) $$\varphi_{{}_{h,i,k}}^{\prime }>\frac{V_{i}-V_{k}}{h_{i,h}-h_{k,h}}\times \frac{100}{w_{h}},\;h_{i,h}>h_{k,h}$$

(31) $$\varphi_{{}_{h,i,k}}^{\prime }<\frac{V_{i}-V_{k}}{h_{i,h}-h_{k,h}}\times \frac{100}{w_{h}},\;h_{i,h}<h_{k,h}$$

where $\varphi_{h,i,k}^{\prime }$ is subjected to the equation:

(32) $$\frac{w_{h}-1}{w_{h}}\times 100<\varphi_{{}_{h,i,k}}^{\prime }<100$$

Supposing the minimum critical value of index weights change with the formula:

(33) $$D_{h}=\underset{1\leq i,k\leq m}{\min}\{|\varphi_{{}_{h,i,k}}^{\prime }|\}$$

Then we define the sensitivity coefficient of weights as the term of:

(34) $$S_{h}=1/D_{h}$$

The $S_{h}$ value represents the mutability of the index weight that contains subjective factors, that is, for the robust purpose, the smaller the better.

In order to calculate the proposed model, we developed a series of programs containing the subroutines of GRA, weighting calculation and sensitivity analysis, etc. The programs were performed by Matlab (v. 8.3.0.532) on a personal computer equipped with an Intel(R) Core(TM) i5-2400 CPU @ 3.10 GHz configuration and 64-bit Windows 7 system.

## 4. A Case Study of Coal-Fired Power Units

### 4.1. Basic Information of Seven Power Units

This section conducts a comprehensive evaluation with the data of seven 600 MW subcritical coal-fired condensing power units in North China in the year 2016, which belong to the North China power grid. The detailed data we used are shown in Table 3. The boiler of unit 1 and unit 2 have the design style of forced circulation and tangential combustion, and the other five boilers are natural circulation and opposed firing style. The fuel of the selected units is bitumite with the supply mode of straight blowing, and all the boilers are using plasma ignition mode. The design efficiencies of the boilers are 93.95% (units 1–2), 93.43% (units 3–4) and 94.36% (units 5–7), respectively. Correspondingly, the design heat consumption rates of steam turbine units are 7762 kJ/kWh (units 1–2), 7773 kJ/kWh (units 3–4) and 8153 kJ/kWh (units 5–7), respectively. The condensers of units 1–4 are cooled by water, and the rest by air.

All the units are equipped with a flue gas purification system, that is, a wet flue gas desulfurization (WFGD) system for SO_2_, selective catalytic reduction (SCR) system for NO_X_ and electrostatic precipitator (ESP) for dust removal. To meet the demand of new ultra-lower emissions of China, these units had been further reformed except units 2 and 5. The reform measures include technologies such as low NO_X_ combustion retrofit coupling with SCR, high-frequency power source retrofit of ESP system and the upgrading of desulfurization system, etc.

### 4.2. Evaluation Results

The comprehensive evaluation was carried out with the hybrid model introduced in Section 3.3. The C-level data is nominalized with the index attribute information (see Table 3), and the BECC weight of C-level indices are obtained listed as the last column in Table 3. With the BECC weights of C-level integrating into the GRA evaluation process, we get the grey relational degree evaluation value vectors of each B-level indices, that is, economic index (B1), environmental index (B2), flexibility index (B3) and power quality index (B4). The four B-level result vector forms a new decision matrix, which is shown in Table 4. The weights columns in Table 4 are calculated based on the B-level decision matrix with the AHP and BECC method, and the combined weight also obtained with the two kinds of weights.

The evaluation result listed in Table 4 shows that the economic performance (B1) of unit 4 is the best and unit 6 is the worst. While for the environmental protection performance (B2), the best one is unit 3 and the worst is unit 2. This is mainly because unit 2 has not been retrofitted with ultra-low emissions measures. The auxiliary energy consumption of environmental protection equipment of the units without ultra-low emission retrofit usually lower than the reformed one, and this will lead to a better economic performance. The flexibility index (B3) of unit 4 and unit 5 have little difference, which is higher than the others obviously, while unit 2 and unit 3 have the similar lower scores. And unit 6 has the best power quality performance, but unit 1 has the worst.

In order to decide the AHP weights of the four B-level indices, experts have been invited to developed pairwise comparison matrix until the consistency check is passed through. Then the AHP weight $w_{j}^{AHP}$ is determined with the consistency ratio of 0.067. The AHP weight in Table 4 reflects that the environmental protection index has attracted the most attention, mostly because of the deterioration of the environmental quality of China in recent years. Flexibility is the second important index of experts’ interested. As mentioned in Section 1, this is closely related to rising proportion of renewable energy generation in the total power generation system. The economic index is the third and the power quality priority is the lowest.

The objective weight of BECC $w_{j}^{BECC}$ shows that it has an analogous priority with the AHP weight. The weight of environmental index in BECC is higher than the weight in AHP. However, the weight of flexibility index is the lowest one, which is incompatible with subjective experience. It also reflects that objective weight only based on the pure data is not an ideal way in sometimes and the subjective knowledge should be added in the evaluation process as an adjustment measure. The integrated weight $w_{j}$ calculated with Equation (21) shows that economic index and flexibility index are having similar value, while the environmental index is still the most important one. The priority of the integrated weight is in consist of AHP weight.

The final evaluation result, i.e., grey relational degree vector of B-level indices can be calculated with the B-level evaluation matrix, and the corresponding integrated weight is shown in Table 4, which is calculated by the model introduced in Section 3.3. The result is:

(35) V=(0.7418, 0.4420, 0.8038, 0.8371, 0.5063, 0.6967, 0.7916)

Finally, the ranking sequence of the evaluated power units based on the grey relational degree from large to small is as follows:

(36) Uint 4≻Uint 3≻Uint 7≻Uint 1≻Uint 6≻Uint 5≻Uint 2

The result represents the comprehensive performance of power units with multiple evaluation criteria. The sorting result shows that unit 4 is the best one according to the proposed multi-level evaluation system. That means, for example, in an ESPGD situation with the seven competitive candidate coal-fired power units, unit 4 will gains the power generating right first.

The excellent performance of unit 4 is due to its economic index which is highest among the seven units, besides, the environmental index and flexibility index performance is higher than the other ones either in a large extent. Unit 2 is the worst one because of the environmental index value is too small, and at the same time, the weight of environmental factor is the most important among the B-level indices. With the similar reason of unit 2, the comprehensive performance of unit 5 is undesirable either, which is just a little better than unit 2.

It is clear that the proposed evaluation framework is different from the typical evaluation method which only considers the economic factor (mainly the net standard coal consumption). If the alternative units are assessed in a typical way, unit 2 is the best, and followed by unit 3, due to the outstanding performance of the net coal consumption rate (C11) index. It is obviously unreasonable in considering the environmental and other aspect evaluation indices. Thus, it is shown that the multi-index comprehensive evaluation is necessary.

The evaluation results also points out the retrofit direction of the units in improving comprehensive performance to achieve a higher score. Taking unit 5 for example, which is sorted as the sixth in the ranking list, the poor score mainly caused by the low performance of economic and environmental factors. However, in considering the flexibility criteria, the performance value is the best one due to the C35 index with a value of 55% adjustable power capacity. That means, if we want to improve the comprehensive performance of unit 5, the economic and environmental performance should be improved by the means such as energy saving retrofits or ultra-low emission reform.

The above discussion about the result shows that the comprehensive evaluation is reasonable and acceptable. The result can point out the weak part of the power generation operation and management of a unit, and the result may provide guidance for the promotion of operating performance.

### 4.3. Sensitivity Analysis Results

The final result of the proposed hybrid evaluation model will be varied when the weights with a possibility of uncertainty. In the process of determining combined weight in the B-level indices with AHP method, the expert preference was integrated as the subjective knowledge. However, the experience may vary with different experts, that means, the result got from the same evaluation model may be changed with different evaluation person. Thus, it is important to carry out weight sensitivity analysis in exploiting the latent evaluation information. Sensitivity results are shown in Table 5 and Table 6, with the analysis method mentioned in Section 3.4.

From Table 5, it can be found that the environmental performance (B2) and electricity quality index (B4) have an obvious effect on the evaluation results. The environmental indicator may lead to seven unit pairs with the inverse order, while the electricity quality may influence six pairs. However, the ranking results of some units are robust, i.e., the result will not be influenced by the B-level weight variation, such as (1,3), (1,4), (1,7), (2,3), (2,4), (3,6), (4,5) and (6,7).

Table 6 illustrated that the environmental performance indicator (B2) has a most sensitive performance, which means the B2 is the easiest indicator to change among the B-level indices. The flexibility indicator (B3) follows with the B2, while the economic index (B1) is the most robust one. The weight changing of index B1 and B2 will cause the raking of unit 5 inverse firstly, with units 2 and 6 respectively. While weight changes of B3 and B4 index bring ranking inverse with the same unit pairs (units 3 and 4).

## 5. Modeling Validation and Discussion

### 5.1. Comparing BECC with Other Objective Weighting Approaches

This section validated the effectiveness of the BECC weighting by comparing it with many other objective weighting methods (M2–M5 in Table 7).

The evaluation values of grey relational degree and unit ranks have been listed in Table 7. The corresponding results were calculated by replacing BECC with M2 to M5 weighing method respectively in the developed hybrid evaluation model illustrated in Figure 3, while the AHP weight remained unchanged during the combing weighting process.

### 5.2. Stability Analysis with Result Uncertainty Caused by the Bootstrap Method

**Figure 4:**
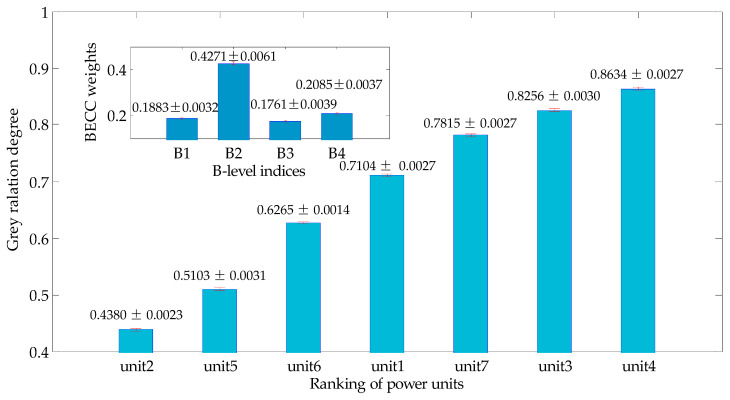
Average value and standard error of result and B-level index BECC weight (with 5000 times cycling).

The evaluation result V may be changed slightly each time while the hybrid model is employed due to the factor of bootstrap technology. However, the difference could be ignorable for the ultimate comprehensive evaluation ranking. To illustrate the details, the whole hybrid model (shown in Figure 3) was repeated for 5000 times to produce a result dataset with a dimension of 7×5000. The result reflects that the ranking of units was not changed over each calculating. Furthermore, we use the standard error of each evaluation result sequence to estimate the uncertainty of the candidate units. Figure 4 shows the statistical averaged results in a form of ranking from small to large, while the standard errors of each unit are presented as a form of error bar with the specific values either.

The uncertainty of each evaluation result (grey relational degree) is primarily caused by the BECC weights (C-level and B-level). Additionally, averaged values and the corresponding standard errors of B-level weight are shown in Figure 4 too. We can observe the uncertainty factors in an input perspective. The result shown in Figure 4 is consistent with the value in Section 4.2. The largest standard error of the B-level combined weights is 0.0061 with index B2, while for the units’ evaluation results, the largest standard error occurs in unit 5 with the value of 0.0031. It is demonstrated that the evaluation results perform a pretty reproducibility and the influence of results uncertainty of the results could be ignorable.

### 5.3. Details of the Resampled Distribution of BECC Process

**Figure 5:**
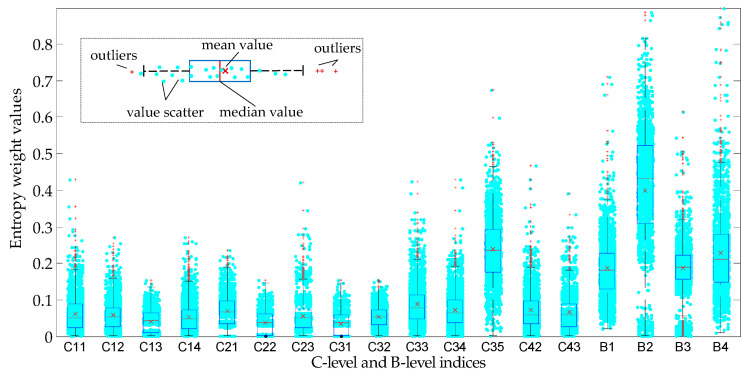
Entropy weight distribution of different evaluation index based on bootstrap resample.

**Figure 6:**
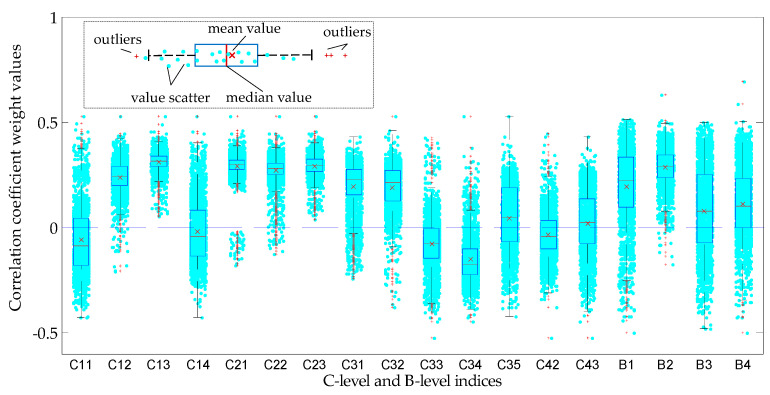
Correlation coefficient weight distribution of different evaluation index based on bootstrap resample.

In order to illustrate the calculation process of the BECC weight, a visualization work about the entropy and correlation coefficient weight was developed. Distributions of entropy and correlation coefficient weight were obtained by resampling the initial data about power units 5000 times. The resampled data were calculated by the steps shown in Figure 2. The distribution of entropy and correlation coefficient weights of each index (C-level and B-level) are shown in Figure 5 and Figure 6 with boxplots and scatters respectively. It can be found that neither the entropy weights nor correlative coefficient weights are symmetrical distribution, especially for the entropy weights, most of which have a long tail on one side (or outlier). It is also demonstrated the normal distribution hypothesis to a small sample is actually unreasonable in sometimes.

### 5.4. Policy Recommendations

The energy conservation and emission conduction for the overall energy system is a complicated integration engineering. The ESPGD method based on multi-index comprehensive evaluation is a promising approach to cope with the question. Some suggestions were proposed here.

1. Accelerating the reforming of the auxiliary services market

The improvement of the flexibility (as the index we selected in the proposed evaluation system) has no direct economic benefit on the unit itself, which just supplies peak shaving service for the power grid and mainly for the renewable energy generation power system. However, the improvement of the flexibility of coal-fired power units is a benefit for energy conservation and emission reduction of the overall power system. Thus a reasonable economic compensation for peak-shaving to the grid will facilitate the positivity increasing of the power plant flexibility performance retrofit. Therefore, the government energy sector should construct the improving measures of the auxiliary service market as soon as possible.

2. Promoting the integrating of multiple indices online monitoring system

The comprehensive evaluation is based on statistical data. In this paper, annual mean values were employed in the case study. It may be made more meaningful by employing online measurement values of the evaluated indices with a month or daily average to accomplish a dynamic evaluation process. However, what the power grid concerns is still the AGC, PFC, and AVC performance indices by EMS online monitoring. The economic indices such as standard coal consumption and environmental pollution indices are not integrated into EMS well in some regional power grid in China. Fortunately, the online environmental parameters supervision system has been completed due to the rigorous air pollution problem in recent years, and the remote standard coal consumption supervision system has been constructed in mainstream energy companies. Therefore, the power grid should make an effort in integrating the economic indicator and environmental protection indicator into the EMS to bring ESPGD into reality.

## 6. Conclusions

In this paper, a multiple index comprehensive evaluation system is constructed for the ranking of coal-fired power units in an ESPGD system. The evaluation system considers four catalog indices covering the economy, environmental protection, flexibility and electricity quality aspects. A hybrid evaluation model integrating GRA and a combined weight method is developed and a novel weighting method BECC is proposed based on an entropy method. A case study is carried out using the hybrid evaluation model and a sensitivity analysis is given too. The following conclusions can be obtained:

1. The multi-index evaluation model is feasible for coal-fired power units. Comparing with the traditional ESPGD method, which is only based on economic and environmental principles, the comprehensive evaluation method introduced in this paper involves the operational flexibility and electricity quality performance indices at the same time. The evaluation results demonstrated that the method is suitable for the practical requirement.
2. The proposed objective weighting method BECC uses bootstrap skills to utilize the population information contained in the selected samples, and BECC also considers the mutual redundancy among indicators by introducing the correlation coefficient into the classical entropy weights. It is proved to be feasible by comparing with some other methods in the literature. Through 5000 times cycles of the whole evaluation model, the statistical result also proved that it is stable with an ignorable uncertainty caused by the stochastic factor in the bootstrapping process.
3. It is an alternative route to integrate a variety of mature methods to get a hybrid model. It is beneficial to assimilate the advantages of each method and reduce the shortcomings. Subjective empirical knowledge is necessary for the assessment process. For example, the AHP could improve the evaluation results which developed just based on the pure data analysis. Additionally, sensitivity analysis of criterion weighting is a good way of exploiting the rich information of the model robustness. The sensitivity analysis in the case study, for example, shows the environmental factor with the biggest sensitivity coefficient.

We believe that the comprehensive evaluation method proposed in the paper is beneficial to the energy saving power generation dispatch (ESPGD) system. The evaluation results could identify the shortage of the various performance of coal-fired power unit and provide the instruction for its improvement.

## Figures and Tables

**Table 1:** Analytical hierarchy process scale.

Importance Intensity	Definition	Meaning (Index X Compared with Y)
1	Equal importance	X is as equally important as Y
3	Moderate importance	X is moderately more important than Y
5	Strong importance	X is strongly more important than Y
7	Very strong importance	X is very strongly more important than Y
9	Extreme importance	X is extremely more important than Y
2, 4, 6, 8		Intermediate values

**Table 2:** Average stochastic consistency rate.

*m*	1	2	3	4	5	6	7	8	9
*RI*	0	0	0.58	0.90	1.12	1.24	1.32	1.41	1.45

**Table 3:** Basic data of the seven power units.

B-Level Index	C-Level Index	Index Unit	Unit 1	Unit 2	Unit 3	Unit 4	Unit 5	Unit 6	Unit 7	Index Attribute ^(^*^)^	C-Level $w_{j}^{BECC}$
B1	C11	g/kWh	315.70	301.64	303.82	312.72	320.99	318.28	328.75	(−)	0.3077
	C12	%	4.72	4.78	4.45	4.23	4.75	5.02	4.44	(−)	0.2565
	C13	T/a	44.54	99.45	37.01	21.15	61.44	54.01	24.22	(−)	0.1994
	C14	kg/(kWh)	0.89	0.47	1.98	0.63	1.55	2.62	1.35	(−)	0.2365
B2	C21	mg/Nm^3^	18.29	84.10	18.12	19.14	79.65	26.10	18.95	(−)	0.3142
	C22	mg/Nm^3^	20.32	116.17	12.93	26.38	47.16	27.15	14.76	(−)	0.339
	C23	mg/Nm^3^	2.54	17.08	2.32	1.51	21.72	3.93	2.65	(−)	0.3469
B3	C31	%	100	99.2	100	100	100	100	100	(+)	0.0695
	C32	-	0.95	0.82	0.97	1.2	1.1	1.08	1.3	(+)	0.0988
	C33	-	1.44	1.39	1.48	1.56	1.53	1.21	1.42	(−)	0.1964
	C34	-	0.83	0.78	0.87	1.02	0.92	1.15	1.2	(−)	0.1364
	C35	%	50	50	50	55	55	50	51	(+)	0.4988
B4	C41	%	100.00	100.00	100.00	100.00	100.00	100.00	100.00	(+)	0
	C42	%	78.6	90.5	80.4	86.8	85.1	89	92.6	(+)	0.5108
	C43	%	4.5	4.8	4	5	4.5	4	4.5	(−)	0.4892
	C44	%	100	100	100	100	100	100	100	(+)	0
	C45	%	100	100	100	100	100	100	100	(+)	0

^(^*^)^ (+) represents benefit attributes, the bigger the better and (−) represents cost attributes, the smaller the better.

**Table 4:** Evaluation value and weights information of the B-level index.

B-Level Index	B-Level Evaluation Matrix							B-Level Weights		
	Unit 1	Unit 2	Unit 3	Unit 4	Unit 5	Unit 6	Unit 7	$w_{j}^{AHP}$ ^(^*^)^	$w_{j}^{BECC}$	$w_{j}$
B1	0.5598	0.7176	0.6699	0.8311	0.4535	0.4103	0.5844	0.1957	0.1908	0.1357
B2	0.9252	0.3553	0.9730	0.9253	0.4227	0.7992	0.9444	0.3788	0.4201	0.5784
B3	0.4730	0.4569	0.4518	0.7647	0.7710	0.5359	0.4965	0.3116	0.1738	0.1968
B4	0.4156	0.5794	0.6782	0.4415	0.4913	0.828	0.7532	0.1139	0.2153	0.0891

^(^*^)^ The consistency ratio CR=0.067<0.1, the consistency check is passed.

**Table 5:** Sensitivity analysis value of B-level weight ($\varphi /\varphi^{\prime }$).

Unit Pairs	B1	B2	B3	B4
(1,2)	-/-	0.4834/82.3213	-/-	-/-
(1,3)	-/-	-/-	-/-	-/-
(1,4)	-/-	-/-	-/-	-/-
(1,5)	-/-	0.4126/70.2603	−0.6871/−353.9947	-/-
(1,6)	-/-	-/-	-/-	−0.205/−237.6440
(1,7)	-/-	-/-	-/-	-/-
(2,3)	-/-	-/-	-/-	-/-
(2,4)	-/-	-/-	-/-	-/-
(2,5)	−0.2661/−201.0095	-/-	-/-	−0.7936/−920.0509
(2,6)	−0.6188/−467.4248	0.4296/73.1515	-/-	-/-
(2,7)	-/-	0.587/99.9612	-/-	-/-
(3,4)	-/-	-/-	0.1146/59.0520	−0.1524/−176.7093
(3,5)	-/-	0.586/99.7833	-/-	-/-
(3,6)	-/-	-/-	-/-	-/-
(3,7)	-/-	-/-	-/-	−0.5704/−661.3729
(4,5)	-/-	-/-	-/-	-/-
(4,6)	-/-	-/-	-/-	−0.611/−708.3858
(4,7)	-/-	-/-	-/-	−0.2554/−296.0747
(5,6)	-/-	0.3243/55.2168	−0.5131/−264.3550	-/-
(5,7)	-/-	0.5337/90.8814	-/-	-/-
(6,7)	-/-	-/-	-/-	-/-

**Table 6:** Sensitivity factors of B-level index.

	B1	B2	B3	B4
$D_{j}$	|−201.0095|	55.2168	59.052	|−176.7093|
$S_{j}$	0.005	0.0181	0.0169	0.0057
Pairs	(2,5)	(5,6)	(3,4)	(3,4)

**Table 7:** Comparative results of different objective weights.

Method	M1		M2		M3		M4		M5	
Method Meaning	BECC		Entropy		M1 without Bootstrap		CRITIC [33]		CCSD [34]	
Evaluation Result	Value	Order	Value	Order	Value	Order	Value	Order	Value	Order
Unit 1	0.7115	4	0.7245	4	0.7121	4	0.7287	4	0.6689	4
Unit 2	0.4390	7	0.4306	7	0.4355	7	0.4537	7	0.4586	7
Unit 3	0.8243	2	0.8197	2	0.8179	2	0.8581	1	0.7922	2
Unit 4	0.8692	1	0.8872	1	0.8760	1	0.8377	2	0.8483	1
Unit 5	0.5084	6	0.5081	6	0.5152	6	0.4913	6	0.5481	6
Unit 6	0.6284	5	0.6261	5	0.623	5	0.6548	5	0.6380	5
Unit 7	0.7808	3	0.7842	3	0.7778	3	0.8131	3	0.7828	3

## Nomenclature

**Abbreviations**	
AGC	automatic generation control
AHP	analytic hierarchy process
AVC	automatic voltage control
BECC	weighting method integrating bootstrap, entropy and CC
CC	correlation coefficient
CCSD	weighing method integrating CC and standard deviation
CI	consistency index in AHP method
CR	consistency ratio in AHP method
CRITIC	criteria importance through inter-criteria correlation
DEA	data envelopment analysis
ELECTRE	elimination and choice expressing reality
EMS	energy management system
ESP	electrostatic precipitator
ESPGD	energy saving power generation dispatch
GRA	grey relational analysis
MCDM	multi-criteria decision making
MICE	multi-index comprehensive evaluation
NDRC	national development and reform commission
PFC	primary frequency control
PROMETHEE	preference ranking organization method for enrichment evaluation
RI	random index in AHP method
SCADA	supervisory control and data acquisition
SCR	selective catalytic reduction
TOPSIS	technique for order preference by similarity to an ideal solution
WFGD	wet flue gas desulfurization
**Symbols**	
ξ	grey relational coefficient
ρ	distinguishing coefficient of GRA
$\hat{\theta }$	estimated parameter based on empirical data
${\hat{\theta }}_{b}^{*}$	estimated parameter based on bootstrap resampled data
$\lambda_{\max}$	maximum eigenvalue of AHP comparison matrix
$\varphi ,\varphi^{\prime }$	absolute and relative minimum weight change
a,x,y,z,p	element of matrix
$a_{j}$	alternative
$\bar{a},\bar{z}$	mean value
$e_{j}$	entropy value
$f_{i}$	indicator vector
max	maximum
min	minimum
r	linear correlation coefficient
A,X,Y,Z	matrix
B	calculation or simulation times
$D_{h}$	minimum critical value of index weight
$\hat{F}\left(x\right)$	empirical probability distribution
${\hat{F}}^{*}\left({\hat{\theta }}^{*}\right)$	bootstrap probability distribution
$S_{h}$	sensitivity coefficient of weight
V,v	grey relational degree vector and element
W,w	weight vector and element
$X_{b}^{*}$	bootstrap resampled sequence
**Subscripts and Superscripts**	
m,n	dimension of matrix
i,j,h,k,b	index of vector or matrix
ref	reference
BECC	based on BECC method
BENT,BCC	based on bootstrap-entropy, bootstrap-CC method
ENT,CC,AHP	based on entropy, CC, AHP method
T	transpose symbol of vector
